# Evaluating Clinician Expectations of mHealth Solutions to Increase Rapid-Screening for HIV and Hepatitis in Migrant Populations in France: Qualitative Study

**DOI:** 10.2196/41861

**Published:** 2023-02-03

**Authors:** Carter Brown, Guillaume Roucoux, Svetlane Dimi, Saleh Fahmi, Raj-Banou Jeevan, Olivier Chassany, John Chaplin, Martin Duracinsky

**Affiliations:** 1 Patient-Reported Outcomes Unit, UMR 1123 Inserm Université de Paris Paris France; 2 Pierre Louis Doctoral School of Public Health Sorbonne University Paris France; 3 Unité de Recherche Clinique en Economie de la Santé Assistance Publique – Hôpitaux de Paris Hôpital Hôtel-Dieu Paris France; 4 International Vaccination and Travel Medicine Center Centre de Santé Familia Sol Creil France; 5 Institute of Health and Care Sciences Sahlgrenska Academy at Gothenburg University The University of Gothenburg Centre for Person-Centred Care Gothenburg Sweden; 6 Service de Médecine Interne et d'Immunologie Clinique Hôpital Bicêtre Le Kremlin-Bicêtre France

**Keywords:** app development, acceptability, mobile health, mHealth, user-centered design, communication barriers, migrants, HIV, AIDS, hepatitis, rapid diagnostic testing, public health, communication tool, screening, language barrier

## Abstract

**Background:**

Migrants underuse screening opportunities for HIV, hepatitis B, and hepatitis C despite elevated risk factors for contracting these infections. Language barriers are an often given as reasons for limiting access to services. Translation and communication apps increase communication and overall patient satisfaction in the patient-provider relationship. In the development and adoption of new technology, expectations play an important role.

**Objective:**

This study aimed to explore health care professionals’ opinions and attitudes regarding their screening practices with migrants and their expectations for a new communication tool that could improve migrants’ screening use.

**Methods:**

In this qualitative study, a purposive (diverse) sampling method was used to invite doctors and nurses who conduct rapid screening tests with migrants from 4 centers of the French Office of Immigration and Integration in 3 geographic regions of France. Semistructured interviews were conducted to survey their opinions on the rapid testing of migrants, the use of telephone interpreters, the concept of health literacy, and their expectations of a new communication tool that could overcome language barriers and promote rapid screening in the new migrant population.

**Results:**

In all, 20 interviews were conducted with 11 doctors and 9 nurses with a median age of 58 (range 25-67) years. Participants favored the integration of an innovative communication tool in the context of rapid screening of migrants. However, there were concerns related to the implementation and added value of the tool while migrants were already reluctant to be screened. Expectations were for a tool that would present information in simplified French or a chosen language but also supports a positive attitude toward screening. Health professionals also expressed the wish that the technology could help with the collection of health data.

**Conclusions:**

Feedback from health professionals provides a better understanding of potential formats, characteristics, functions, content, and use of an innovative, digital method to communicate with migrants with limited French proficiency. Findings contribute to the conceptual development of an electronic app and its implementation within the ApiDé study, which aims to validate a digital app to address language barriers to increase the use of screening among migrants with limited French proficiency in France.

## Introduction

In 2018, nearly 6200 people were diagnosed with HIV in France. Among these individuals, more than half (3224/6200, 52%) had never been tested for HIV in their lifetime, 81% (5022/6200) were injection drug users, and 65% (4030/6200) were from sub-Saharan Africa [1]. A French survey from blood donors estimated the prevalence of hepatitis B virus (HBV) to be 53.1% (250/471) among migrants from endemic regions while attributing nosocomial exposure as the leading factor for hepatitis C virus (HCV) [2]. Furthermore, many HIV-positive migrants in Europe acquire their infections after migration [3].

The French health authority (*Haute Autorité de Santé*) guidelines for annual screening recommends that people who have multiple partners from endemic regions, notably sub-Saharan Africa and the Caribbean, should be screened regularly for HIV, HBV, and HCV [4]. Furthermore, recommendations suggest that these 3 tests should be conducted at the same time [5]. Between 2017-2020, a total of 21,133 migrants were tested for HIV, HBV, and HCV at the French Office of Immigration and Integration (*Office Français de l'Immigration et de l'Intégration* [OFII]) [6].

In France, legal migrants must undergo a medical examination as part of the administrative process to obtain a residence permit at OFII. During this consultation, health professionals (HPs) propose a free-of-charge, rapid antigenic and/or antibody diagnostic test (*test rapide d’orientation diagnostique* [TROD]) for HIV, HBV, and HCV with results in 20 minutes or less. Individuals who test positive are oriented for follow-up care, which is also provided free of charge.

Barriers to accessing screening services in the migrant population in France have been previously documented. HPs frequently cite linguistic barriers as major obstacles negatively impacting the acceptability of the diagnostic testing [7]. A recent study in immigrants in Canada found that language barriers interfered with preventative and screening services and ultimately lead to poor health outcomes [8]. Additionally, a study investigating knowledge, behavior, and practices related to HIV and sexually transmitted infections among migrants from sub-Saharan Africa living in Germany found that German language proficiency was one factor associated with knowledge about German HIV policies and HIV testing [9].

A systematic review investigating language barriers in migrant health care found that translation apps enable better communication in the patient-provider relationship and reduce overall consultation times [10]. Another study demonstrated that a mobile translation app contributed to the use of interpretation services and resulted in a high level of satisfaction among HPs [11]. The benefits that mobile health apps could provide to HPs’ communication needs have not been investigated in relation to virus testing in migrants who have limited French proficiency (LFP).

Innovative methods to reach vulnerable and migrant populations are needed to increase the acceptability of TROD [12], but there is a lack of evidence on how HPs experience language barriers in this context. Knowledge of HPs’ expectations is fundamental to the development of new technology and plays an important role in determining the rate at which it will be adopted [13]. Therefore, it is important to explore expectations in the early phases of the new technology’s life cycle when there is uncertainty regarding performance [14].

This research is part of the STRADA study that started in 2017 to determine the acceptability of TROD from both the migrants’ and HPs’ perspectives [7]. The objective of this qualitative study was to explore how HPs envisioned a hypothetical new tool that could help increase communication with migrants with LFP to explain the importance of rapid screening at OFII. We believe that by engaging HPs in the conception of a future app that the app will be better made and that HPs would be more likely to use said app.

## Methods

### Population and Setting

This qualitative study was conducted in a population of doctors and nurses who work in 4 OFII centers in France (Lyon, Nice, Cergy, and Montrouge). Each center employed more than 5 HPs on site and had varying volumes and origins of migrants. A purposive (diverse) sampling method was used to include equal numbers of doctors and nurses, male and female, who regularly offer rapid screening test to migrants and who previously participated in the STRADA screening study. All interviews were conducted face to face in the informant’s workplace.

### Interviews

Semistructured interviews were conducted using an interview guide (Multimedia Appendices 1 and 2). This guide was created with themes that emerged from previous interviews with migrants, data that have not yet been published, and with reference to literature and expert opinions. The interviewers (SF and RBJ) explored the HPs’ experience with migrants outside of the OFII context, medical visits with migrants at OFII, rapid testing of migrants, the use of telephone interpreters, the concept of health literacy, and the HPs’ opinion on the creation of an electronic tool to promote rapid screening of migrants with LFP. This paper solely covers the last theme; future articles will appear on the other topics.

### Data Collection

In all, 20 in-person interviews were conducted from May 15 to October 20, 2019. The interviews lasted roughly 30 (range 15-44) minutes. Interviews were audio recorded. Data were collected according to Consolidated criteria for reporting qualitative research (COREQ) guidelines [15] (see Multimedia Appendix 3). Inclusions continued until the interviews perceived data saturation.

Interview recordings were transcribed verbatim (Amir Haourara, Florent Lidec, Catherine Boivin, and RBJ) and then coded (Anis Harbi, CB, GR, MD, Olivia Rousset Torrente, RBJ, and SF) to facilitate thematic analysis using a General Inductive Approach using the methodology developed by Thomas [16]. Triangulation coding was conducted with open-source Sonal software (Alex Alber, Université F. Rabelais [Tours]). The coding process was developed over time following several meetings among the research team (CB, GR, MD, and SF) and then analyzed (GR).

### Ethics Approval

The study was approved by the Inserm Ethics Independent Committee (00003835, protocol 2016/43NI) and then registered with French data protection authority (2008669). Verbal consent was obtained from each participant prior to interviews.

## Results

### Sociodemographic Characteristics

Participants included 11 doctors and 9 nurses who conducted medical examinations at OFII. In all, 14 (70%) participants were female. Their median age was 58 (range 25-67) years, with a median of 25 (range 2.5-40) years of professional experience and a median of 3 years (range 1 month to 22 years) of working at OFII. Combined, the participants conducted medical examinations in 9 languages. Only 1 (5%) participant spoke solely French. Details are displayed in Table 1.

**Table 1:** Health professionals’ sociodemographic characteristics (N=20).

Characteristic		Participants, n (%)
**Sex**		
	Male	6 (30)
	Female	14 (70)
**Age group (years)**		
	20-29	1 (5)
	30-39	1 (%)
	40-49	3 (15)
	50-59	9 (45)
	60-69	5 (25)
**Localization**		
	Center 1	6 (30)
	Center 2	5 (25)
	Center 3	8 (40)
	Center 4	1 (5)
**Position**		
	Medical doctor	11 (55)
	Nurse	9 (45)
Having a health care speciality^a^		6 (30)
**Seniority as a health professional (years)^a^**		
	<10	1 (5)
	10-19	6 (30)
	20-29	4 (20)
	30-39	7 (35)
	40-49	1 (1)
**Seniority at OFII^b^ (years)^a^**		
	<1	3 (15)
	1-9	9 (45)
	10-19	4 (20)
	≥20	2 (10)
Having another job outside of OFII job^a^		16 (80)
**Previous professional experience (before OFII)^a^**		
	With migrants	10 (50)
	HIV, HBV^c^, or HCV^d^ prevention activities	8 (40)
**Native language^a^**		
	French	15 (75)
	Not French	5 (25)
**Number of foreign languages spoken^a^**		
	0	1 (5)
	1	7 (35)
	2	9 (45)
	3	3 (15)

^a^Missing data for 1 participant.

^b^OFII: Office Français de l'Immigration et de l'Intégration (French Office of Immigration and Integration).

^c^HBV: hepatitis B virus.

^d^HCV: hepatitis C virus.

### Thematic Analysis

**Figure 1:**
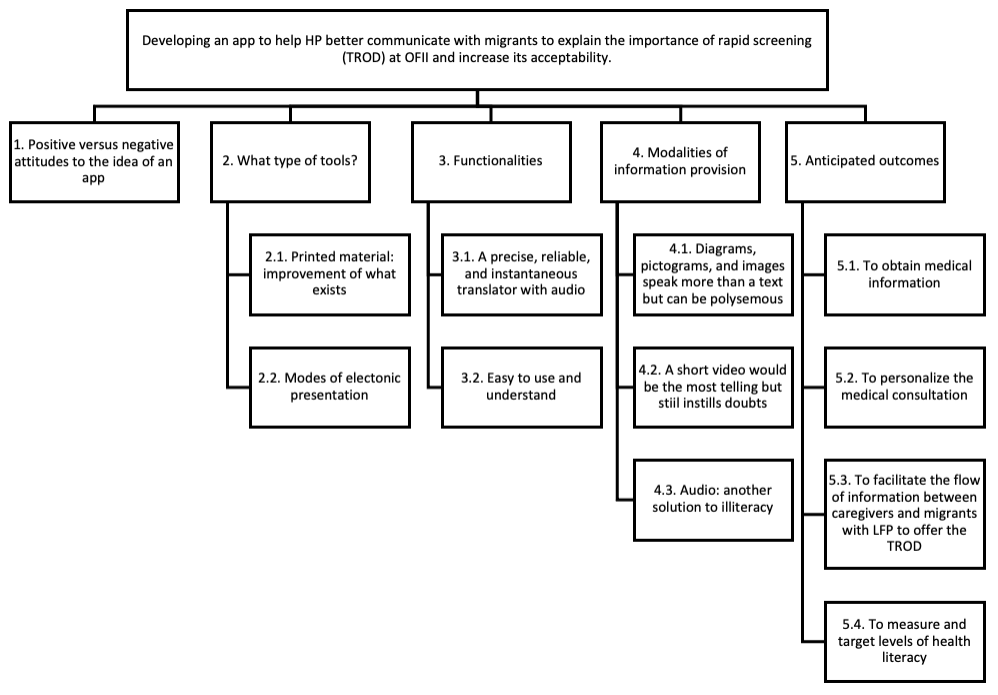
Thematic tree. HP: health professionals; LFP: limited French proficiency; OFII: Office Français de l'Immigration et de l'Intégration (French Office of Immigration and Integration); TROD: test rapide d'orientation diagnostique (rapid diagnostic test).

In all, 5 major themes and 11 subthemes were defined during the thematic analysis, detailed in the following diagram (Figure 1).

### Positive and Negative Attitudes Toward an App

All HPs reported having used (at least once, in their private life) a translation app such as Google Translate (the most cited). Most of them used one at OFII, in absence of a better solution, by only formulating wording-simplified close-ended questions. Some noticed mistranslations, which caused no overall misunderstanding though. They reported that migrants with LFP spontaneously used translation apps too. HPs were not opposed to a tool that would help them do their job that would also benefit their patients, “I’m in favour [of a tool] because it is for the patient’s benefit” (male doctor). One doctor thought that an app could be used by doctors to “give their opinion and explain [to the patient] what our objective is [as an HP] in the context of rapid screening” to better communicate with the patient (male doctor).

However, some HPs who were concerned about the effectiveness of an app to actually improve the uptake of screening because of the migrant with LFP’s preconceived notions of the screening process. “Not understanding the added value” or “a lack of trust” no matter how good the app is were mentioned. “For [migrants with LFP], there is no interest. They have already got it into their heads ‘I don't want to do it,’ or else ‘I’m going to do it,’ but it’s not the health benefit that motivates them” (female nurse).

In addition, it was suggested that a new tool would be difficult to implement “because people are on the move, left and right [during the medical consultation at OFII], uh...I don’t know when they would have time to use it” (female nurse). It was also expressed that instead of a new tool, it would be better to revise current screening practices (female nurse).

### What Type of Tools?

#### Printed Material–Improvement of What Exists

One participant suggested that when planning a new tool, it should begin with revising the existing texts that are used: “translate our proposition [to conduct a rapid test]. To have that information already translated for the LFP…it’s super important” (female nurse).

Currently, HPs at OFII reference a binder containing documents translated into 13 languages, with standard sentences used to ask sociodemographic and health history questions. Several participants were satisfied with a paper-based system. One participant, who relies on this printed material, said that he preferred “printed documents.”

For me, a good tool is a printed document [translated] into the patient’s mother tongue...For me, I get out the document...there you go. It’s all I need. The LFP reads it and...while doing so, I try to follow what they are reading because I know [what’s written]. Basically, I know what the questions correspond to...they answer, and then it saves me from having to rely on Google translate or calling an interpreter. It’s quick.”

male doctor

Some centers have created their own documents so that additional languages can be offered; therefore, practices differ from center to center. Another participant said that a new tool could simply be an improvement to the existing paper-based system. The new tool could therefore be “a sheet of paper” (female doctor) or “a poster” (female nurse) placed in the waiting room, since “some [migrants] have long wait times” (female nurse).

Other participants are resistant to the idea of the new tool having a paper format because it assumes that the patient is literate, as two participants explained: “Some can’t even read, so...there’s a disconnect” (female nurse).

#### Modes of Electronic Presentation

Informants spoke of how a new app might augment existing technology that they already have the habit of using. The new tool could be used “over the phone” (female doctor) or “on a tablet” (female nurse), as well as “A tool on the internet, on the computer” (male doctor), such as “Google Translate” (male doctor). Another mentioned “artificial intelligence and technology” (male doctor).

### Functionalities

#### A Precise, Reliable, and Instantaneous Translator With Audio

Participants wanted the device to be precise and reliable, “the formulation must be really refined” (male doctor), because the HPs need a “support that is reliable” (female nurse) and trustworthy.

The main expectation of HPs is that a new device would be a “translator” (male doctor). HPs would appreciate a translator that has an audio function and works simultaneously with their speech. This would make it possible to solve the illiteracy of certain migrants: “We would speak, and [the tool] would translate at the same time, for people who can and who cannot read, there would be the audio” (female nurse). This would guarantee the confidentiality of information (compared to a third party, such as a professional or informal interpreter):

You just put your language in, and then when you are speaking, it translates immediately, the person understands, they speak...Well, I will answer you frankly. The ideal for me is simultaneous translation, perfect. There you go, if you want efficiency for work and confidentiality during the consultation, that’s all.

male doctor

#### Easy to Use and Understand

HPs imagined a simple tool that is both easy to use and understand: “simple words. It shouldn’t ask too many questions. The [migrant] must also be able to understand...it has to be easy to use, practical for the consultation.” (male doctor). Another participant wanted the tool to translate “according to the levels” of knowledge of the migrant with LFP (female nurse).

### Modalities of Information Provision

#### Diagrams, Pictograms, and Images

HPs suggested adding “small diagrams” (female nurse): “why not include images?” (male doctor). Visual communication was seen as “a supplement; it can...help” (female doctor). One participant stressed that “it's about the drawings” (female nurse). Another recalled that “there are people who have an educational level which is not [enough to read]...there are quite a few, [so] yeah, [there’s a need] for pictograms, drawings” (male doctor). However, one expressed doubt about images having divergent cultural meanings: “a different interpretation of a pictogram, they [the migrants] are not always perceived in the same way” (female doctor). Although images were considered to be useful, there was concern that they were polysemous.

#### Video

A video was considered to be a more effective way to transmit a message compared to plain text, audio, or pictograms: “it would be the most effective” (female nurse). Several participants would like the same characteristic: a “little video” (female nurse) of “short duration” (female doctor), nothing longer than “3 minutes” (female nurse).

However, one participant doubted the added value of video: “In respect to a video, when you are infected, you will already have a document to read...would an image or video add anything in addition to the text? Hm…” (male doctor). Another participant expressed the potential difficulty of using video in an OFII waiting room: “a video, in my opinion, would be the most informative. But in fact, putting that in place, I’m not sure it’s very easy” (female nurse). Therefore, a short video was considered the best was to present information but challenging to implement.

#### Audio, Another Solution to Illiteracy

The use of “a voice” or audio was mentioned by 2 participants: “It’s not bad [the audio], like an exhibition [at a museum]” (female nurse). Another participant saw the benefit as a means to overcome illiteracy: “Perhaps with audio, yeah, because we still have the issue with people who can't read” (female nurse).

### Anticipated Outcomes

#### To Obtain Medical Information

Several doctors mentioned numerous questions that they would like to see in the tool. These were questions that they need to pose concerning vaccinations, surgical operations, tattoos, previous and frequent illnesses, procreation, and risks taken—similar to “a classic medical exam” (male doctor).

#### To Adapt to the Patient’s Needs

The tool was also envisioned by HPs to extend their work beyond the screening process. One participant envisaged a tool that could provide immediate feedback during the consultation that could be personalized according to “the person in front of us, if he says to us: ‘I have been operated on,’ it [the app] informs us, and we easily understand” (male doctor), enabling the HPs to be able to conduct a more thorough risk assessment. Several HPs had noticed a lack of knowledge about the medical interventions that the patients had experienced and wanted the new tool to adapt to this lack of knowledge:

If the person says, “I had surgery. Well, they cut my stomach open.” “What exactly did they have done?” There are people who don’t understand what kind of interventions they’ve had...It seems useful to me [to inform people] of the basic things at least, to provide information.

male doctor

Another participant took up the same theme by proposing that the tool help the HP educate the migrant with LFP: “I could explain the mode of transmission!” (female nurse). Another participant suggested that the tool encourages migrant with LFP to educate themselves with the HPs they will meet in consultations: “‘If you want more information, the person in front of you can help you’” (female nurse).

#### To Facilitate the Flow of Information Between the HP and Migrant With LFP to Offer the TROD

The tool was described as a potential aid to the medical relationship in that it could be used before the migrant is asked about the TROD, it would say to the migrant: “‘Here, we are looking for such things, and here is what [we offer you]’...then they know [what] we are looking for” (male doctor). It would “give them [the migrant] confidence from the start [of the migrant’s arrival at OFII].” One participant expected “that [the tool] would not disturb the climate of the medical consultation” (male doctor), “if there is something specific, well, we would ask questions” (male doctor).

#### To Measure and Target Levels of Health Literacy

Several participants talked about a new tool offering opportunities for data collection that were not possible within the current protocol. Principally, this was the inclusion of a health literacy test that could be integrated into the tool. Participants reported that “it might be interesting for us to know” the migrant’s level of understanding (female nurse). This test would contain questions such as “Do you know these different diseases, hepatitis B, hepatitis C, HIV? Do you know how they are transmitted?” (male doctor). The tool would then report the migrant’s answer to the HPs who would be able to adapt their presentation to the patients’ level of understanding.

## Discussion

### Principal Findings

Despite doubts of a digital communication tool and ingrained habits using printed translated materials, participants favored the integration of an innovative digital tool to enhance communication with migrants with LFP in the context of rapid screening. Although some participants found current printed materials effective, this communication method is unidirectional and thus does not promote a dialogue between the patient and provider. Furthermore, HPs spoke of the perceived benefit of using translated and culturally adapted multimedia content to better communicate with their patients and enhance the consultation experience for all parties, thus requiring a digital solution. These positive expectations will attract HPs to the innovative tool once developed and will play a crucial role in the mobilization of resources for its’ successful implementation [17].

A systematic review Investigating the use of electronic tools to help increase testing in migrants with LFP, conducted by our research team, found that translation apps provide better communication with HPs and have a high acceptability of use [10]. In terms of a new tool, our research found that HPs spoke most frequently of an easy to use (Figure 1, subtheme 3.2), accessible app with multiple features, including visual (Figure 1, subthemes 4.1-4.2) and audio (Figure 1, subtheme 4.3) components along with an accurate, reliable, and instantaneous translation (Figure 1, subtheme 3.1); data collection; adaptive content; and interpretation functions (Figure 1, subthemes 5.1, 5.2, 5.3, and 5.4).

A cross-sectional study comparing patient-provider communication with IT-mediated communication versus face-to-face communication found the same level of effectiveness, although patients prefer face-to-face communication with their provider [18]. Although we did not study the patients’ perspective, participants expressed interest in a new tool that could be tailored to the medical consultation (Figure 1, subtheme 5.2) and adapted to the patient’s needs. The patients’ perspective, however, needs to be further explored to understand their perceptions of mixed communication methods, which would include digital and face-to-face communication during the same consultation.

During the interviews, HPs spoke of their need for a tool that could obtain medical information from their patients, facilitate communication to offer the TROD, and measure patients’ level of health literacy, which would then provide educational material to patients. A systematic review found that touchscreen apps could help patients with limited health literacy better understand medical information and provide education on medical treatments [19]. Furthermore, a prototype to support patient-provider interaction in chronic HIV care found that patients want an app that is easy to use and intuitive while meeting confidentiality and security standards [20].

The use of artificial intelligence can provide user-targeted messages to increase the effectiveness of communication and education [21]. Such technology can reach wider and often harder-to-reach audiences than traditional means of communication. One artificial intelligence chatbot deployed in India to encourage conversations on sexual and reproductive health found that the app was an educationally beneficial tool for reaching vulnerable audiences [22].

An app, in the patient’s native language, could help educate patients about the benefits of being screened while at the same time helping the HPs to propose and conduct rapid screening. This would increase the patient’s understanding of HIV, HBV, and HCV including modes of transmission and risk reduction practices. Interfacing with an app could also create a more comfortable context to learn about topics such as sex and high-risk situations than if the HPs interview them on these subjects.

With the increase in mobile technologies in the health sector, an app would be an innovative mobile health approach to increase the screening rate of HIV, HBV, and HCV in an effort to achieve national and international objectives.

### Study Strengths

This study is the first of its kind in the French context. Interviews with both nurses and physicians who conduct medical exams and rapid screening tests at different centers allowed us to gain a better overall understanding of how language barriers effect medical consultations. It also provided us with insight into what HPs want from a communication tool to overcome language and cultural barriers. We found that a better-adapted communication intervention could help HPs overcome language barriers with migrants with LFP and ultimately, increase screening rates.

### Limitations

A limitation of our study is that this research was conducted in only one context and therefore not representative of migrant screening throughout France. Although we included centers in both the Paris region and in other areas of France, there are more than 30 OFII centers, and immigration is not homogenous throughout France.

### Conclusion

Our research allows us to better understand the expectations of health care providers for new technological solutions. These expectations are crucial to the development and adoption of the technology. We have explored the potential format, characteristics, functions, content, and use of a new technology to communicate with migrants with LFP. In terms of an app, we found positive expectations and support from HPs to develop and use an app in the patient-provider relationship to overcome language and cultural barriers.

This information will be used to develop an app and implement the ApiDé study [23], which aims to validate a communication app in an attempt to address language barriers and, ultimately, increase screening rates of migrants with LFP in France.

## Appendix

Multimedia Appendix 1 French interview guide.

Multimedia Appendix 2 English interview guide.

Multimedia Appendix 3 Consolidated criteria for reporting qualitative research (COREQ) guideline checklist.

## Abbreviations

COREQ: Consolidated criteria for reporting qualitative research
HBV: hepatitis B virus
HCV: hepatitis C virus
HP: health professional
LFP: limited French proficiency
OFII: Office Français de l'Immigration et de l'Intégration (French Office of Immigration and Integration)
TROD: test rapide d'orientation diagnostique (rapid diagnostic test)
