# Supplementary material for: Evaluating Clinician Expectations of mHealth Solutions to Increase Rapid-Screening for HIV and Hepatitis in Migrant Populations in France: Qualitative Study
Source: JMIR Hum Factors. 2023 Feb 3;10:e41861. doi: 10.2196/41861 (PMC9938434; doi:10.2196/41861)
Supplement: Multimedia Appendix 3 [file humanfactors_v10i1e41861_app3.pdf]

COREQ guideline checklist

**Domain 1: Research team and reflexivity**	**Personal characteristics**	
	1. Which author(s) conducted the interview?	SF conducted 17 interviews<br>RBJ conducted 3 interviews
	2. What were the researcher's credentials?	CB: PhD candidate in Public Health<br>GR: PhD candidate in Sociology<br>SF: MD, MPH, MBA<br>RBJ: Master of Psychology<br>MD: MD, PhD
	3. What was their occupation at the time of the study?	CB: employed as researcher at INSERM UMR1123, Sorbonne University School of Public Health and Epidemiology<br>GR: employed as researchers at INSERM UMR1123<br>SF: employed as researcher at INSERM UMR1123<br>RBJ: intern at INSERM UMR1123<br>MD: team leader of PROQOL INSERM UMR1123
	4. Was the researcher male or female?	CB: male<br>GR: male<br>SF: male<br>RBJ: female<br>MD: male
	5. What experience or training did the researcher have?	CB: training in qualitative methods for public health<br>GR: training in qualitative methods for ethnology & sociology<br>SF: training in qualitative methods for public health<br>RBJ: training in qualitative methods for psychology<br>MD: training in qualitative methods for public health
	**Relationship with participants**	

	6. Was a relationship established prior to study commencement	No relationship was established prior to study commencement
	7. What did the participants know about the researcher?	The participants only knew that the researchers were public health researchers.
	8. What characteristics were reported about the interviewer/facilitator?	We reported the following characteristics:<br>SF: Is trilingual (Arabic, French, and English). He worked for 3 years in Montreal within the *Médecin du Monde* clinic for migrants. He introduced himself as a researcher in public health.<br>RBJ: Is bilingual (French and Tamoul) with a professional-level of English. She introduced herself as an intern in public health.
**Domain 2: Study design**	**Theoretical framework**	
	9. What methodological orientation was stated to underpin the study?	Inductive General Approach & Thematic Analysis
	**Participant selection**	
	10. How were the participants selected?	From a list of available doctors and nurses at the OFII offices with who conduct screening based on the availability of interviewers
	11. How were the participants approached?	Official contact through the selected OFII office with the help from the centres' directors
	12. How many participants were in the study?	20
	13. How many participants refused to participate or dropped out? Why?	None
	**Setting**	
	14. Where was the data collected?	At the OFII offices in Montrouge, Cergy, Lyon, and Nice – all located in France.
	15. Was anyone else present besides the participants and researcher?	No one else was present during the interviews, which were conducted in an office with the door closed. The researcher and participant were the only individuals present.

	16. What are the important characteristics of the sample?	Participants' characteristics are reported in the "Results – sociodemographic" section
	**Data collection**	
	17. Were questions, prompts, guides provided by the author? Was it pilot tested?	An interview guide with both closed (sociodemographic) and open-ended questions
	18. Were repeat interviews carried out? Details	No interviews were repeated
	19. Did the researcher use audio or visual recording to collect the data?	All interviews were audio-recorded, with prior participant consent
	20. Were field notes made during and/or after the interview or focus group?	Field notes were taken during all interviews
	21. What was the duration of interviews or focus groups?	Interviews lasted between 15 to 44 minutes (average: 26 minutes)
	22. Was data saturation discussed?	The research team discussed data saturation
	23. Were transcripts returned to participants for comments and/or correction?	Transcripts were not returned to participants
**Domain 3: Analysis and findings**	**Data analysis**	
	24. How many data coders coded the data?	7: AH, CB, GR, MD, ORT, RB, SF
	25. Did authors provide a description of the coding tree?	Partially. It only displays themes covered by the article
	26. Were themes identified in advance or derived from the data?	Themes were derived from data (general inductive approach)
	27. What software, if applicable, was used to manage the data?	Sonal (2.1.41)
	28. Did participants provide feedback on the findings?	No
	**Reporting**	
	29. Were participant quotations presented to illustrate the themes/findings? Was each quotation identified?	Participants' quotations are presented to illustrate findings.
	30. Was there consistency between the data presented and the findings?	Yes
	31. Were major themes clearly presented in the findings?	Yes
	32. Is there a description of diverse cases or discussion of minor themes?	Yes
